# INTERGENERATIONAL SOLIDARITY AND FILIAL PIETY PERCEIVED BY OLDER ADULTS IN RURAL CHINA

**DOI:** 10.1093/geroni/igad104.3065

**Published:** 2023-12-21

**Authors:** Wencheng Zhang, Merril Silverstein, Ying Xu

**Affiliations:** Syracuse University, Syracuse, New York, United States; Syracuse University, Syracuse, New York, United States; Syracuse University, Syracuse, New York, United States

## Abstract

Filial piety—the obligation of adult children to serve and honor older parents—is a fundamental value in Chinese society. This study explores how dimensions of intergenerational solidarity influence parents’ evaluations of their children’s filial piety. Data derive from 1473 respondents reporting about 4000 relationships in the 2021 wave of the Longitudinal Study of Older Adults in Anhui Province China. Strength of filial piety was assessed for each adult child along with other aspects of the relationship. Random effects ordinal logistic regression predicted parents’ perceptions that their children fulfilled expectations of filial piety based on geographic proximity, financial support, and emotional support. Findings indicate that parents consider children who live with them as most filial, and they view residentially independent children who live farther away as less filial. However, financial support from more distant children raises perceptions that these children are meeting expectations of filial piety. In addition, having more frequent contact with distant children strengthens perceptions of them as being filial. Results suggest that weak filial piety perceived by older parents of their more geographically distant children in rural China—an area characterized by high rates of internal migration—is compensated by financial transfers and frequent communication. As research shows that filial piety perceptions are important to the well-being of older adults in Chinese populations, threats to its decline due to forces of modernization and any adverse consequences may be overcome by forms of intergenerational solidarity that involve contributions of money and time by children to their older parents.

